# Discovery of Rare Phenotypes in Cellular Images Using Weakly Supervised Deep Learning

**DOI:** 10.1109/ICCVW.2017.13

**Published:** 2017-10-29

**Authors:** Heba Sailem, Mar Arias–Garcia, Chris Bakal, Andrew Zisserman, Jens Rittscher

**Affiliations:** 1 Department of Engineering Science, University of Oxford, Parks Road, Oxford, OX1 3PJ, UK; 2 Institute of Cancer Research, 237 Fulham Road, London SW3 6JB, UK

## Abstract

High-throughput microscopy generates a massive amount of images that enables the identification of biological phenotypes resulting from thousands of different genetic or pharmacological perturbations. However, the size of the data sets generated by these studies makes it almost impossible to provide detailed image annotations, e.g. by object bounding box. Furthermore, the variability in cellular responses often results in weak phenotypes that only manifest in a subpopulation of cells. To overcome the burden of providing object-level annotations we propose a deep learning approach that can detect the presence or absence of rare cellular phenotypes from weak annotations. Although, no localization information is provided we demonstrate that our Weakly Supervised Convolutional Neural Network (WSCNN) can reliably estimate the location of the identified rare events. Results on synthetic data set and a data set containing genetically perturbed cells demonstrate the power of our proposed approach.

## 1 Introduction

High-throughput microscopy generates vast amounts of data capturing phenotypes in healthy and abnormal cellular populations on routine bases. Automated detection and classification of these phenotypes in large imaging data sets are crucial for advancing biomedical studies and identifying novel therapeutic targets. However, analysis methods for these data sets are still lagging behind as these methods generally rely on strong supervision which is not feasible with the deluge of generated images. Furthermore, both pixel-level annotations and object-level annotations can be biased by the experience of an individual observer. Therefore, there is a great need for generalizable learning methods that can learn to predict and highlight the differences between samples from weakly labeled (i.e. image-level labels) training data.

### Why rare phenotype detection?

In many cellular imaging classification problems, we are interested in only a small subpopulation of the cells in an image such as cancerous or mitotic cells [2, 14]. Additionally, cellular populations are highly heterogeneous and often only a subpopulation of cells responds to perturbations resulting in an abnormal phenotype [13]. Consequently, only a small percentage of the overall cell population is of interest. It is not clear a priori which features discriminate such rare phenotypes from the remaining cell population. Ideally, such features should be learned from the data in order to generalize to the wide spectrum of cellular phenotypes.

### Why weakly supervised learning?

With the rapid technological advances in large-scale experimental biology methods, the labeling and manual assessment of visually complex phenotypes become problematic. However, information that relates to experimental conditions or compound treatments is readily available and can be used as image-level annotations. The ability to classify and localize phenotypically different cells between cell populations is crucial for discovering or validating treatment effects. Once a consistent phenotypic difference, even a weak one, between biological samples has been identified, experimentalists can validate the detected difference.

**Figure 1:**
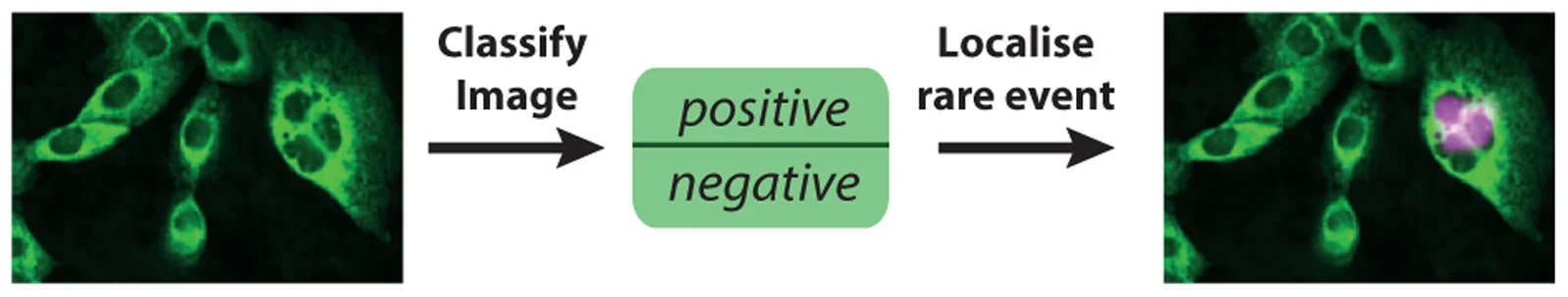
Workflow for weakly supervised rare phenotype detection. The WSCNN network is trained on image-level classes that indicate the absence of a rare or abnormal phenotype (negative class) or the presence of such phenotype (positive class) in the image. In the shown example the abnormal phenotype is multinucleate cells. Our WSCNN is able to detect and localize the multinucleate cell even though it is surrounded by uninucleate cells.

We address the challenge of detecting rare cellular events by leveraging (i) the power of Convolutional Neural Networks (CNNs) in learning features that consistently discriminate between different classes and (ii) the ability of max-pooling operation to select the most consistent features across different layers regardless of their location. There is evidence that CNNs that are trained to predict image-level labels can successfully localize objects associated with different classes [11]. We further illustrate here that our Weakly Supervised CNN (WSCNN) is able to detect and localize different instances of an abnormal phenotype even when it is surrounded by very similar objects in intensity and texture. Our learning pipeline is illustrated in Figure 1.

The details of the classification framework are presented in Section 2. Using a synthetically generated data set, we demonstrate how our approach results in a robust and reliable detection of rare events. A data set containing 1600 images of both normal and multinucleated cells is used to evaluate the performance of the proposed method in a challenging and biologically relevant setting. The details of the data sets and CNN training are described in Section 3. The results are discussed in Section 4. Summary and conclusions are presented in Section 5.

### Related work

In most studies, weakly supervised learning is formulated as a multiple-instance learning (MIL) task where the image is considered as a bag of regions [1, 3]. The image is labeled positive if at least one region contains the object of interest (rare phenotype in our case) and negative if no region in the image contains such object. Such learning paradigm does not only overcome the burden of providing object-level annotations but also can be crucial in studies where generating detailed annotations is not feasible. Recent studies demonstrated that CNNs are able to classify and localize objects given only weak image-level annotations [1, 8]. In contrast to our problem, the methods proposed in these studies have been evaluated on data sets, such as PASCAL VOC, where the objects often occupy most or a reasonably large region of the image. Furthermore, the different object classes are usually very distinct in their color and structure.

On the other hand, cellular images contain many instances of cells that have very similar morphology and appearance. The difference between classes includes morphological changes [13], changes in protein abundance, or differences in protein localization [9]. There has been an increasing interest in utilizing the power of CNNs in classifying various cellular phenotypes. However, most studies employ deep learning only as a part of the image analysis pipeline such as segmentation or feature extraction [4, 9]. For example, Parnamaa *et al*. [9] applied CNN-based learning to the classification of protein subcellular localization in yeast cells using cropped images of presegmented cells.

Their results illustrate that CNN-learned features outperform carefully hand-crafted features and shallow classification methods such as random forests [9]. Similarly, Kandaswamy *et al*. [4] applied CNNs and transfer learning to classify the effects of drugs with known mechanism of action on cells. Again they only used deep learning as feature extractors from presegmented cell images. Given the segmentation task, Ronneberger *et al*. [10] demonstrated the successful application of CNNs to cell and tissue image segmentation. Extending a similar approach, Wang and collaborators [12] present an architecture that can detect a number of different cellular subtypes. However, all of these approaches either rely on manually generated object-level labels or presegmented images which do not address the need for fully automated classification systems.

A capable CNN architecture for the classification of cellular images from image-level labels has been proposed by Kraus *et al*. [5]. But given the high frequency of the events they are detecting, their work does not investigate rare event classification. In contrast, we are aiming to detect objects or phenotypes that are rare and only occupy a small region of the image from weak and noisy annotations.

## 2 Classification and localization framework

Our aim is to classify the presence or absence of a rare phenotype (e.g. multinucleate cells) given only image-level labels. We formulate this task as a binary classification problem where images with positive labels have at least one cell with a rare phenotype while images with negative labels have no such cells 1. The input to the CNN is images that contain around 20-100 cells and the corresponding class labels (i.e. positive or negative for the rare phenotype). Most positively labeled images have few phenotypically different objects, i.e multinucleate cells. We train the network to predict whether an image contains a rare phenotype. Before the concrete design of the architecture is presented, we discuss the two components that are crucial for the detection and localization of rare events.

### 2.1 Feature selection using max pooling

Our work is motivated by the intuition that the maxpooling operation searches for the best discriminative features in an image by propagating only the pixel values with the highest local score to the subsequent layers. Therefore, they can be regarded as signal amplifiers where at each layer only the best scoring pixels are selected for further learning. Based on this intuition we hypothesize that a CNN utilizing a max-pooling operation following convolutional layers should be able to classify images where different classes are very similar except for very small regions that have an aberrant appearance. Probabilistically, feature values that discriminate rare events from the rest of the population have extremely low probabilities. Therefore, using average pooling which sums the evidence from different features would swamp the discriminative feature values. This would make the rare event detection almost impossible, which we confirmed experimentally.

#### Global max pooling

In order to summarize the pixel scores from the entire feature map, global max-pooling is applied over all the pixels in the final convolutional layer. This is in line with Oquab *et al*. [8] who utilized global max pooling to perform weakly supervised classification and localization. The final convolutional layer is composed of an output unit for each class where each unit can be regarded as a detector of that class. Let *a*_*k*_(*x, y*) represent the activation of the class detector *k* at a spatial location (*x, y*). Then, performing a global max pooling for class detector *k* produces a single score (one pixel value) for each class *A*_*k*_ = *max*(*a*_*k*_(*x, y*)). These scores are passed to the softmax loss function. Importantly, max-pooling aggregation function allows CNNs to detect discriminative features regardless of their location.

### 2.2 Visualization and localization of rare phenotypes

Although we do not provide any localization or segmentation information during training, we show that the network can implicitly learn to localize rare events. If the network classifies an input image as positive, regions which contributed significantly to the classification score can be used for visualizing the detected differences between classes. Simulated data is used to validate the localization accuracy. In addition, our approach provides a qualitative way of analyzing the phenotypic difference between different cell populations, such as the difference between normal and genetically perturbed cells.

Different methods [7, 11, 15] have been proposed for visualizing and understanding CNN representations. The error backpropagation-based visualization method proposed by Simonyan *et al*. [11] is extended here to visualize rare events. Given an image *I* we aim to generate a saliency map that displays the contribution of each pixel site x to the classification score *S*. This is achieved by back propagating the CNNs class specific scores *S*_*c*_ in the penultimate layer through the trained network. At each layer, the rank of each pixel can be approximated by the derivative of the linear function *y* = *w*^*T*^ *I* + *b*. This can be regarded as the Jacobian map with respect to specific class predictions of image *I*. The originally proposed method [11] produces highly pixelated and noisy saliency maps. We apply Gaussian smoothing and local non-maximum suppression to generate more smooth segmentation (see Figure 4 and 5).

### 2.3 CNN architecture

Given the relatively small size of the objects of interest, a modest number of layers is sufficient to achieve good performance. Cellular images contain many instances of very similar but relatively independent objects. Consequently, convolutional layers that identify locally discriminative features are more appropriate to this problem than Fully Connected (FC) layers that attempt to model the relationship between detected features or objects. Oquab *et al*. [8] also report a similar strategy for efficient learning in weakly supervised learning tasks.

**Figure 2:**
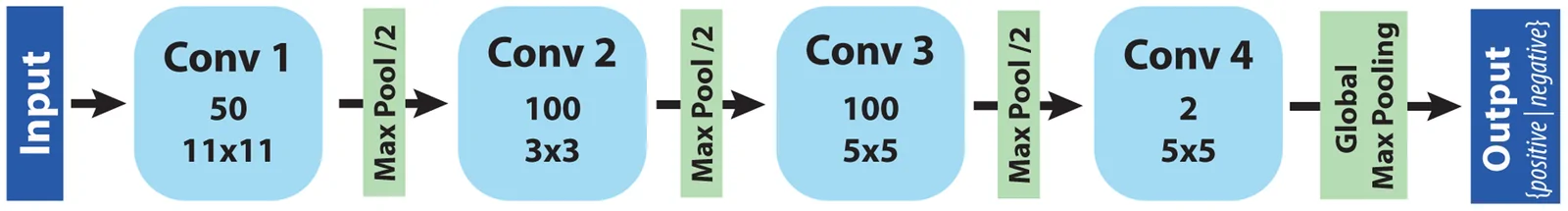
Weakly supervised CNN architecture for classifying rare phenotypes. The figure illustrates an architecture which utilizes 4 convolutional layers with 50, 100, 100 and 2 features respectively. Max-pooling downsampling of size 2×2 is applied following each convolutional layer except for the last layer where a global max-pooling is applied.

The input to our WSCNN is grayscale images and the output is whether the image belong to the positive or negative class. We employ four convolutional layers starting with a filter of size 11×11, and reducing the filter size in the subsequent layers to 3×3, 5×5, and 5×5 as in [6] (Fig. 2). Apart from the last convolutional layer, all convolutional layers are normalized using batch normalization, and equipped with rectification linear units (ReLU). A padding of 1×1 pixel is applied to convolutional layers 3 and 4. Each convolutional layer is followed by max-pooling subsampling. The final convolutional layer contains two convolutional filters, one for each class. As motivated before, global max pooling is used after the final convolutional layer to aggregate information from the entire image. We also investigated the performance of a deeper architecture as discussed in Section 4.1.

## 3 Data sets and CNN training

### 3.1 Data sets

#### Simulated data set

For the purpose of validation, we simulated data so that we can control the occurrence of rare events. To mimic the subtle difference between normal and abnormal cells we introduced circles as the outlier subpopulation to a collection of ellipses. A data set of 4000 images (2000 images per class) was generated by defining an equally spaced grid of 48 cells (6 rows × 8 columns). A variable number of objects between 30 and 48 were drawn at the defined positions and shifted by a random number between 1 and 20 pixels. The dimensions of the object were also generated randomly as a number between 15 and 20 pixels. The rotation angle of each ellipse was generated randomly. In the positive class we replaced 10% of the ellipses with circles (Sim. Data 1) and hence the classifier is called *E100_C10*. The resulting images size is 210 pixels × 280 pixels. The data was then split into three equally sized partitions for training, validation, and testing. In order to test the robustness of our approach to the frequency of the rare event, we simulated another data set where 20% of the objects in the positive class are circles (Sim. Data 2).

#### Multinucleate cells data set

A set of 1600 images of both wild-type and genetically perturbed MCF10a breast cells was used to test the method in a relevant biological study. Cells are stained with a cytoplasmic stain (anti-PDI). The wild-type cells are normal and expected to have one nucleus per cell. By knocking down cytokinesis regulating genes these cells become multinucleated if they go into mitosis as they fail to divide after nuclei replication. The genes that were knocked down are ECT2, AURKB, and RACGAP1. Here, only meta-data on the experimental conditions but no pixel-level annotations are available. However, as cells are heterogeneous we expect to have few images in the negative class with multinucleate cells. 800 images containing between 5 and 30 cells each were acquired for each class at 40x resolution. Data were split into three equally sized training, validation, and test sets. Training images were generated by concatenating four randomly selected raw images from each class in a 2×2 grid. To further augment the images, this process is repeated eight times for each raw image. The resulting images were downsampled into 356×462 pixels.

### 3.2 CNN training

We trained an independent model for each data set from scratch using stochastic gradient descent. The hyperparameters are: momentum = 0.9, weight decay = 0.0005, and mini-batch size=50. Learning rate is set to 0.0001 and lowered by a factor of 10 as the error plateaus. We normalized the images by subtracting the mean of all images in the data set.

### 3.3 Data augmentation

Both data sets are augmented extensively in order to make the CNN classifier robust to transitions, especially to rotation and scaling. We employed four augmentation strategies: (i) horizontal and vertical flipping, (ii) rotation with = 20° to 20° or = 160° to 200°, (iii) translation of ±40 pixels, and (iv) scaling by ±50 pixels. We found that extensive augmentation is critical for CNN to accurately and robustly learn the occurrence of the rare events.

## 4 Experiments and results

### 4.1 CNN classification results

#### Simulated data

Our CNN achieves 99.62% accuracy on Sim. Data 1 where only 10% of the objects in the positive class images are circles (Table 1). This corresponds to 2-5 circles per image as we vary the number of drawn objects and augment data by rotation and shifting. To test whether the *E100_C10* CNN is sensitive to the frequency of the circles in the population, we evaluated its performance on images that have 80% ellipses and 20% circles in the positive class (Sim. Data 2). *E100_C10* model trained only on Sim. Data 1 can correctly predict that images with 20% circles belong to the positive class (accuracy = 99.5%) (Table 1).

**Figure 3:**
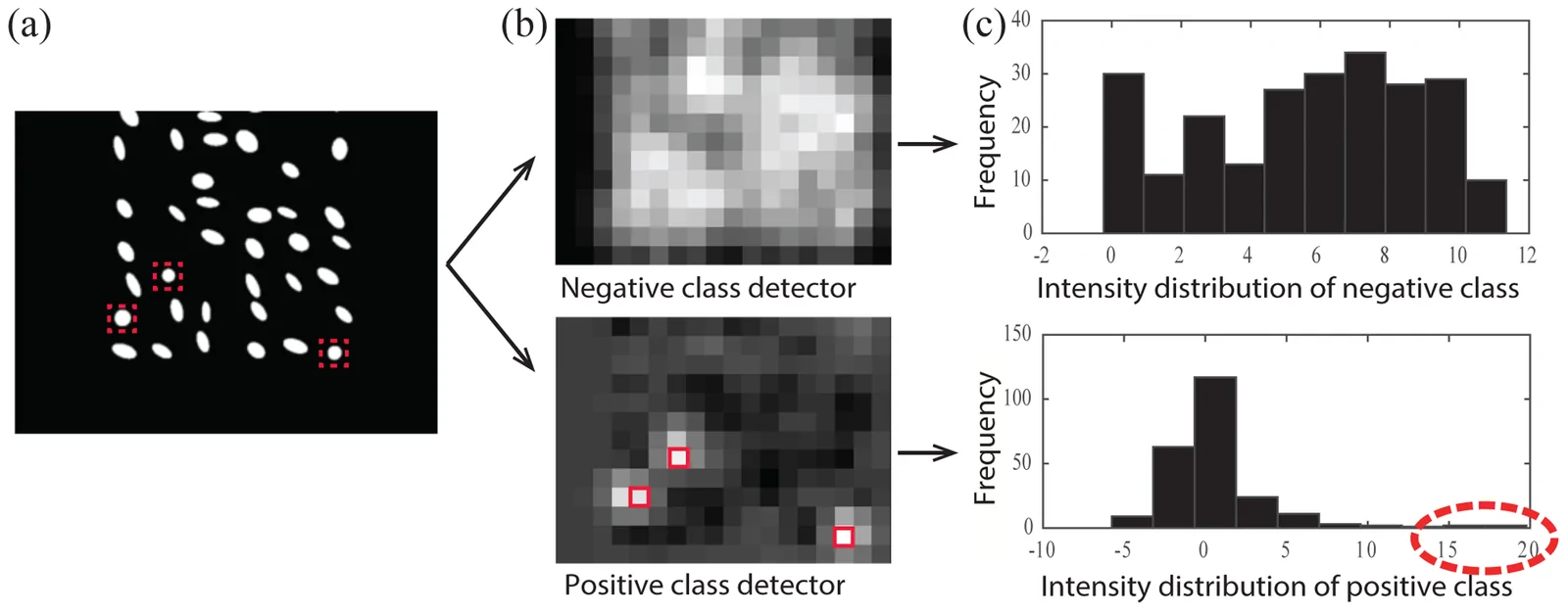
Input images and representation of E100 C10 CNN model features. (a) Input image. Circles are indicated in red to aid the reader but not provided during training. (b) Output feature maps of the last convolutional layer for the negative and the positive class. (c) The intensity distribution of the feature maps in (b). Pixels with the highest intensity are indicated in red.

To understand how CNNs utilizing max-pooling can efficiently classify rare events, we investigated the feature maps learned by the *E100_C10* CNN. Figure 3 shows the feature maps of the class detectors (last convolutional layer) of the *E100_C10* CNN. When the input image contains few circles, then only a few pixels in the positive class feature detector have higher activation values than the negative classs activation values (indicated in red in Fig. 3 (b-c). The pixels with the highest activation in the positive class detector correspond well to the position of the circles in the input image. Using global max-pooling operation, the feature map for each class is reduced to 1×1 pixel value reflecting the maximum activation of that class. This explains how our CNN can accurately predict the presence of rare events.

To further confirm the importance of the max-pooling operation to the rare event detection we replaced the global max-pooling with average-pooling and trained the network from scratch. A CNN with global average pooling operation (WSCNN-Avg) completely failed in classifying images with rare event (accuracy = 51.03%) (Table 1). This is rather expected as the value of the discriminative pixels of the weak phenotype (*e.g*. pixels indicated in red in Fig. 3 (b)) are lost when averaged with the values of all the other pixels in the learned feature maps. Furthermore, we tested adding an FC layer instead of global max-pooling after the last convolutional layer. FC layers have proved beneficial in classification problems where it allows learning the spatial relationship between different pixels in the feature maps while max-pooling discard such spatial information. A CNN utilizing an FC performed significantly worse than CNNs with global max pooling (Table 1). These results confirm the crucial role of using max-pooling downsampling to efficiently classify rare events, a finding that has also been reported by Oquab *et al*. [8].

#### Cell data

To demonstrate the usability of our CNN architecture in real applications, we tested the performance of our method in classifying images of multinucleated cells given only image-level annotations. The CNN for processing the cell images (input image size: 356×462) is identical to that of simulated data except for the filter size in the second convolutional layer is increased to 7×7. Our WSCNN predicted images that have multinucleate cells in the test data set with high accuracy of 90.27% (Table 2). Because the cell images are bigger and more complex than the simulated data, we also tested using a deeper architecture of six convolutional layers. The accuracy improved slightly to 91.89% by increasing the number of convolutional layers (Table 2). Importantly, segmented saliency maps confirm that WSCNN was able to detect and localize the multinucleate cells despite many other uninucleate cells also being in the image (Fig. 5).

### 4.2 Location prediction results

**Figure 4:**
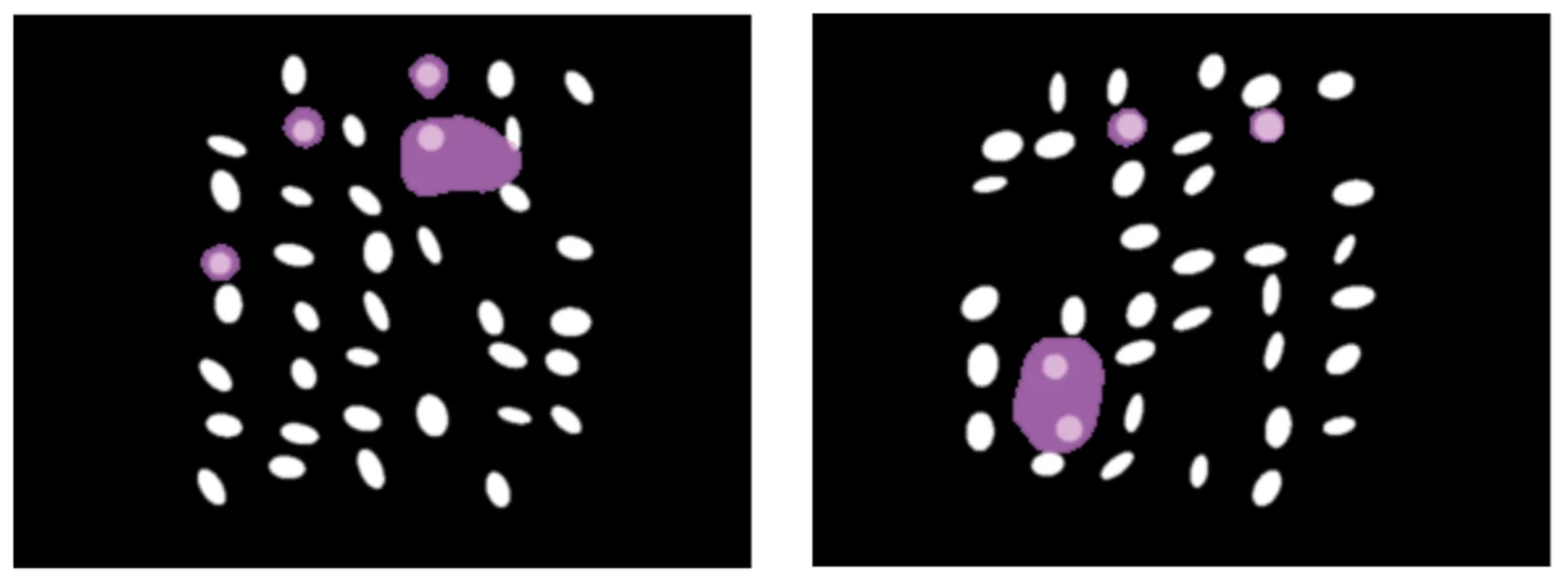
Segmented saliency maps for the simulated data. The maps (shown in purple) were generated by a single backpropagation pass through WSCNN followed by thresholding and smoothing. WSCNN correctly detects and localizes the presence of a rare event of circles in the positive class although only image-level labels were used for training.

Given that our trained CNN achieves excellent classification performance of predicting the presence of a rare event, we sought to investigate its performance in localizing these rare events. The simulated data set has been designed to facilitate this analysis. Using the test data, we generate segmentation masks by thresholding and smoothing the saliency maps generated by backpropagation of classspecific scores given a certain input image (Fig. 4).

#### Localization metric

To measure the localization power of our CNN architecture, we introduce a metric that assesses CNN performance in (i) detecting the different instances of the rare event as well as (ii) detecting the extent of the object. A circle location is considered to be correctly predicted (true positive), if it’s bounding box intersects with the segmentation mask by at least 90%. Otherwise, the prediction is counted as false negative. Similarly, a prediction for an ellipse in the positive class images is counted as false positive if it’s bounding box intersects with the segmentation mask by more than 90%. The WSCNN localization accuracy, precision, and recall scores are calculated based on these numbers.

Using this localization metric, our weakly supervised CNN achieves high accuracy in detecting the different instances of the rare event as well as their extent (Table 3). The accuracy of the CNN on the test data is 99.5% which is comparable to the classification performance confirming that the proposed architecture successfully learns the weak phenotype. The precision is slightly lower than the recall indicating that a few ellipses are confused for circles. However, these ellipses have lower confidence in the score maps and therefore their values are overridden with the higher scoring pixels through the max-pooling operation. These results confirm that the proposed WSCNN is not only able to predict the occurrence of the rare event but also implicitly learns the location of such event.

**Figure 5:**
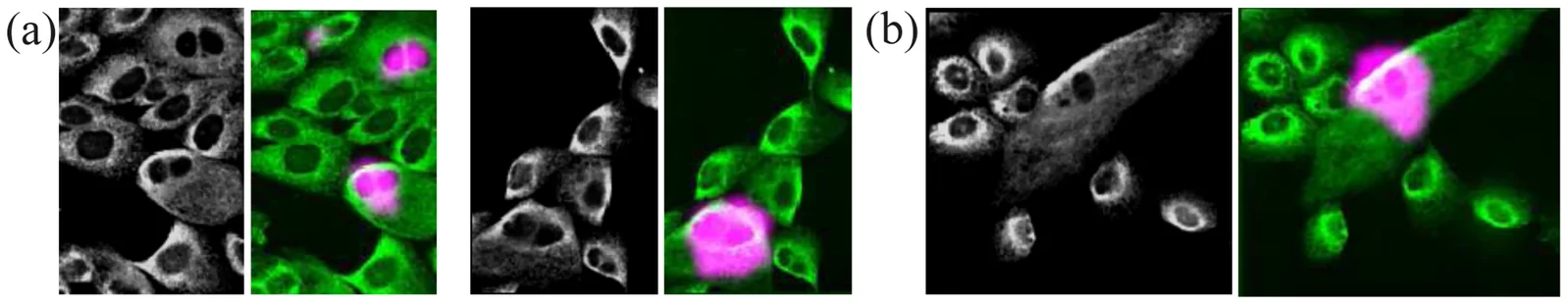
Segmented saliency maps for the multinucleate cells. (a) The saliency maps (shown in magenta) confirm that WSCNN trained using image-level annotations can detect and localize the difference between the positive class and negative class, which is the presence of a few multinucleate cells in the positive class. (b) An image that belongs to the negative class based on the experimental metadata is correctly predicted to have a multinucleate cell.

For the cell images, the only information that is being provided are the experimental conditions (i.e. wild-type versus genetically perturbed for cytokinesis regulating genes). We qualitatively assess that the network is able to identify that the phenotypic difference between the negative and positive class is the presence of multinucleate cells in the positive class (Fig. 5). The network correctly predicts that some of the negative class images are mislabeled and actually do contain multinucleate cells (Fig. 5 (b)). Importantly, this confirms that our CNN architecture is robust to noisy labels which is highly valuable in many biomedical classification problems.

## 5 Discussion & Conclusions

Here we propose a generalizable approach for the discovery of weak phenotypes or rare events from cellular imaging data. Unlike traditional feature-based methods, deep learning provides an attractive approach to this problem as it does not rely on a particular feature set but rather learn such features from the data. To our knowledge, the application of CNNs to this problem is new and has not been applied to either natural images or other biomedical imaging studies. The results demonstrate that our weakly supervised CNN architecture can accurately classify images which contain a small cell subpopulation of an abnormal phenotype.

We conclude that WSCNN can detect rare events by explicitly searching and selecting local features that are consistently discriminative of the negative versus positive class through max-pooling. Furthermore, our CNN is able to localize the evidence regions for the phenotypic difference. While we achieve near perfect performance on the simulated data, the performance degrades as expected on the cellular data. Here, image noise and artifacts cause significant difficulties. Furthermore, the annotations of the cellular images are based on meta-data specifying the experimental conditions rather than pixel-level ground truth. Consequently, there are few images that are mislabelled. Importantly, many of these images were classified correctly by our CNN which predicted the right phenotypic class indicating the robustness of our method to noise.

The principle goal of this work is the development of a generalizable approach for discovering weak phenotypic differences between experimental conditions from large-scale imaging studies. Our lack of prior knowledge on how cells respond to certain treatments motivates why this particular approach holds a lot of promise in the context of phenotypic screening. We controlled the frequency of the rare event by simulating data where 10% or 20% out of 30-50 objects have a different phenotype. Our results show that the WSCNN provides consistent results that are independent of the frequency of the rare event. We content that our framework is highly beneficial in studies where the phenotypes resulting from thousands of pharmacological treatments of cells are unknown a priori. In this frame-work, CNNs can be used for discovering phenotypic differences resulting from perturbations, rather than learning from imaging data sets with a perfect ground truth. Furthermore, saliency maps can be used to qualitatively assess the discovered phenotypes. Such a flexible framework can have a great impact in discovery-based studies and lead to an objective and systematic identification of novel phenotypes and therapeutic targets.

## Figures and Tables

**Table 1:** Classification results on simulated test data. WSCNN-Max: CNN with global max-pooling, WSCNN-Avg: CNN with global average pooling and WSCNN-FC: CNN with FC instead of global max pooling. Sim Data 1 contains 10% circles while Sim. Data 2 contains 20% circles in the positive class images. The results for Sim. Data 2 is based on the *E100_C10* CNN that was trained only on Sim. Data 1.

Data set	CNN Config.	Accuracy	Precision	Recall
**Sim. Data 1**	WSCNN-Max	99.62%	99.26%	99.92%
	WSCNN-Avg	51.03%	2.19%	1.45%
	WSCNN-FC	86.73%	99.40%	74.60%
**Sim. Data 2**	WSCNN-Max	99.5%	99.2%	99.7%

**Table 2:** Classification results on multinucleate cells data. Number of convolutional layers are indicated in parentheses.

CNN Config.	Accuracy	Precision	Recall
**WSCNN-Max (4)**	90.27%	88.24%	90.20%
**WSCNN-Max (6)**	91.89%	88.97%	93.65%

**Table 3:** Localization prediction scores of WSCNN on the Sim Data 1 test set based on segmented saliency masks. True positives are defined as circles that their bounding boxes intersect with the segmentation mask by at least 90% in the positive class images. Similarly, false positives are defined as ellipses that their bounding boxes overlap with the segmentation mask by at least 90% in the positive class images.

Localization accuracy	Precision	Recall
99.995%	93.00%	97.19%
